# Lessons learned responding to the COVID-19 pandemic

**DOI:** 10.1017/ice.2020.293

**Published:** 2020-06-11

**Authors:** Anna C. Sick-Samuels

**Affiliations:** 1 Department of Pediatrics, Johns Hopkins School of Medicine, Baltimore, Maryland; 2 Department of Hospital Epidemiology and Infection Control, Johns Hopkins Hospital, Baltimore, Maryland

*To the Editor—*The coronavirus disease 2019 (COVID-19) pandemic entered our lives without significant notice. Only a few weeks elapsed from hearing of the first reports of a novel viral pneumonia cluster in Wuhan, China to being faced with a full-blown pandemic affecting our own communities. I had recently joined the faculty as a pediatric infectious diseases physician and associate hospital epidemiologist when COVID-19 rapidly usurped our attention. Within a few days in early March, my attention shifted from defending research grant proposals, contemplating central-line bloodstream infection prevention, and completing clinical notes to assisting the hospital respond to COVID-19 full time. I found myself working with colleagues to rapidly draft new protocols, leading multidisciplinary meetings, and helping the hospital navigate the many new challenges presented by this pandemic. From the perspective of a junior faculty member, I would like to share some of the lessons I have learned from this crash course in emergency response. Though framed by the COVID-19 pandemic, these lessons will remain applicable after COVID-19 has left the main stage.

1. Listen and let people finish. Sometimes it is most important to stop talking and listen. Especially when we are using teleconference or zoom calls and we cannot actually see each other’s facial expressions, try to let people finish their sentences. Not only because people have important things to say but also because we feel valued when we are heard. Listening is a way to show respect.
2. Repeat important messages. When the issues and guidance are changing rapidly, we need to repeat the important messages. Repeat to your immediate circle or leadership. Repeat to the broader audience. Repeat with written communications. Repeat with pictures or videos. Repeat in person with individuals.
3. Anxiety is a strong motivator. Especially when working with thoughtful and detail-oriented colleagues. When people do not feel comfortable with a plan, it will not proceed as anticipated and individuals may modify the plan to align with their concerns. Decisions need to consider both physical safety and emotional safety.
4. Brainstorming is different than decision making. As many of us grappled with making decisions for a novel situation with limited data without a precedent to build on, brainstorming discussions occurred on widely attended forums. Sometimes ideas were interpreted as decisions, which can lead to confusion. For those listening, it is important to flag brainstorming from decision points. As a listener, it is important to ask for clarity when it is unclear whether a decision was made.
5. Kindness is vital. Recognize where people are coming from, and respond kindly, even if you do not agree. Everyone showing up is present because they care and are willing to chip in. We must be kind to each other and build together.
6. Disagreement is an opportunity. Many times disagreement ultimately leads to better plans. It may take time and patience to revisit decisions and consider different angles. However, these discussions typically lead to a better plan, or an improved communication of the same plan.
7. Be honest about uncertainty. This is a lesson learned during clinical training- that one should not pretend to know the answer when one does not know. Sometimes communicating uncertainty is extremely difficult but it is important to distinguish opinions from facts.
8. Fatigue is real. Physical or emotional. We are all human. It is necessary to self-recognize and protect one’s own health, especially when others are counting on you. When we are so tired we cannot think clearly, or so drained we cannot be kind, we are no longer effective leaders, colleagues, parents or partners.

I am tremendously impressed with the response and effort for COVID-19, and I feel fortunate to be facing this crisis surrounded by such thoughtful and dedicated colleagues. The COVID-19 crisis has likely led many of us to reflect on why it is we wake up, leave our family and come to work. So for everyone doing just that, continuing to contribute to keeping our patients and colleagues safe, thank you. Your dedication to patients and the well-being of our communities is amazing. As a result of COVID-19, we will be stronger and more flexible to face the next challenge.

